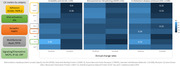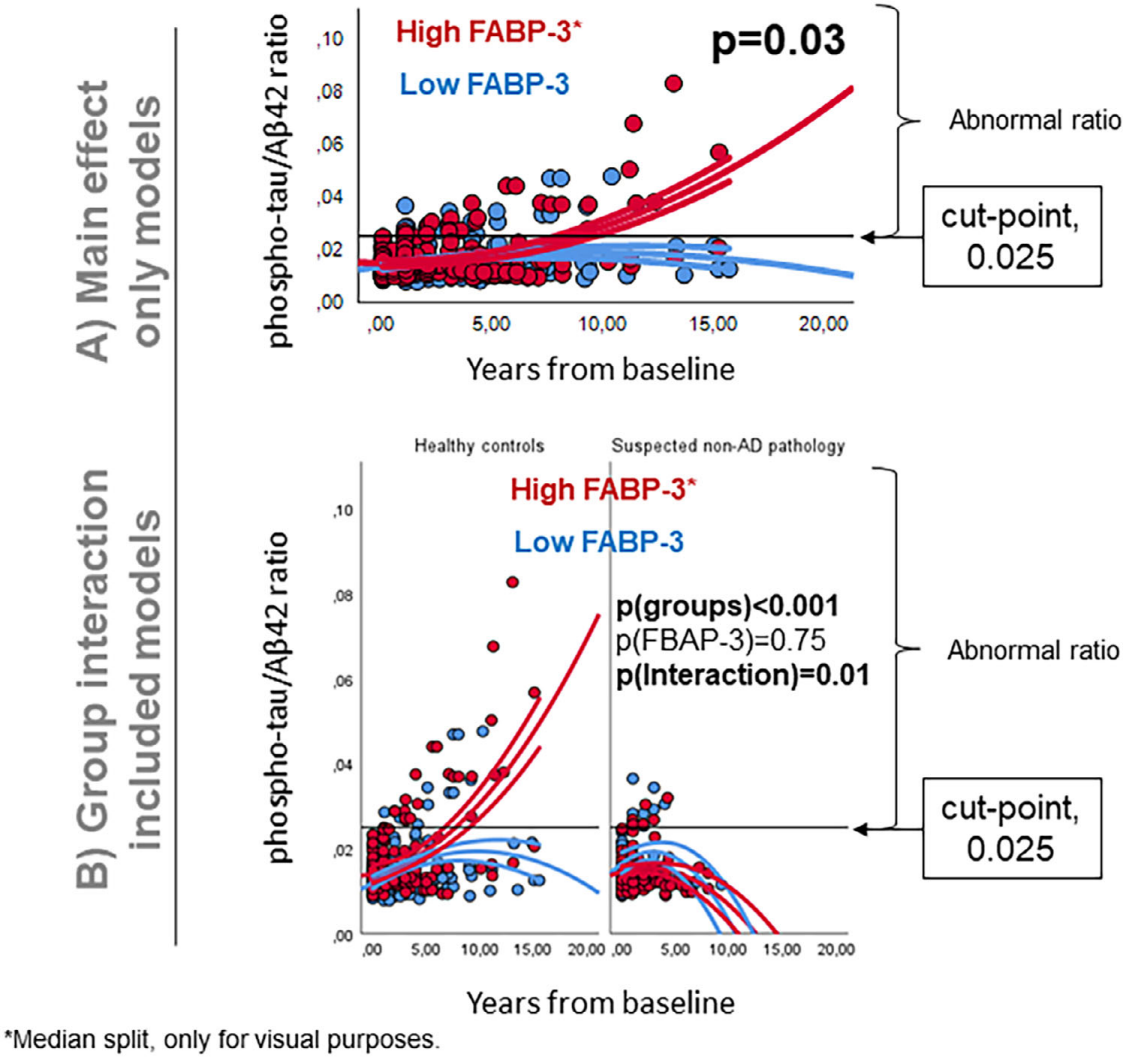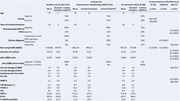# Neuroinflammation and synaptic markers relate to trajectories of core biomarkers in Alzheimer’s Disease continuum

**DOI:** 10.1002/alz.091169

**Published:** 2025-01-09

**Authors:** Ersin Ersoezlue, Julian Hellmann‐Regen, Alzheimer’s Disease Neuroimaging Initiative

**Affiliations:** ^1^ Charité – Universitätsmedizin Berlin, corporate member of Freie Universität Berlin and Humboldt‐Universität zu Berlin – Institute of Psychiatry and Psychotherapy, Berlin, Berlin Germany

## Abstract

**Background:**

Mechanisms of cellular senescence affecting neuroimmune responses, homeostasis, and synaptic functioning associate with core Alzheimer’s disease (AD) biomarkers, i.e., pathological aggregation of β‐amyloid (Aβ) and tau protein, while their longitudinal associations remain unclear.

**Method:**

The study cohort included participants from the Alzheimer’s Disease Neuroimaging Initiative study, which consisted of n=65 healthy controls (HC, mean years of every follow‐up visit 4±3), n=41 suspected non‐AD pathology (SNAP, mean years of every follow‐up visit 4±3), and n=198 AD continuum (ADC, mean years of every follow‐up visit 3±3) who underwent lumbar puncture at the baseline and available data for multiplex based immunoassay panel for the a priori selected analytes in cerebrospinal fluid (CSF). We derived adjusted annual change rates as slopes from separately conducted linear mixed models with Aβ42 or phosphorylated‐tau181 (p‐Tau) dependent variables, including age, sex, APOE‐ε4 status and year from baseline.

We tested the correlations between annual change rates and nine CSF analytes (listed by category in Figure 1). Significance level was defined as alpha=0.05, and the Bonferroni‐correction method was used for multiple testing. We also explored the associations of an incident abnormality of p‐Tau/Aβ42‐ratio with CSF markers using Cox regression models and subsequently tested interactions.

**Result:**

The demographic, genetic, clinical and biomarker levels are presented in Table 1 for each study group, showing that only FABP‐3 levels were altered in ADC compared to HC and SNAP. In both HC and ADC groups, markers for glia and cell adhesion were negatively correlated with annual change of Aβ42 and positively with p‐Tau, respectively (Figure 1A‐C). Specifically in ADC, higher FABP‐3 was associated with an increasing trend in p‐Tau, while FRTN with a decline in Aβ42 (Figure 1C). In the exploratory cox regressions, higher FABP‐3 levels were associated with conversion to abnormal p‐Tau/Aβ42‐ratio status over the up to ∼15 years follow‐up period (Figure 2).

**Conclusion:**

Our results suggests that CSF biomarkers based on cell adhesion, glial activity and synaptic function can contribute to a more precise identification of individuals at‐risk for AD and prediction of trajectories of established AD biomarkers. Moreover, identifying related biological stages of AD can guide the development of preventive approaches targeting these pathways.